# Gene expression profiling reveals novel TGFβ targets in adult lung fibroblasts

**DOI:** 10.1186/1465-9921-5-24

**Published:** 2004-11-30

**Authors:** Elisabetta A Renzoni, David J Abraham, Sarah Howat, Xu Shi-Wen, Piersante Sestini, George Bou-Gharios, Athol U Wells, Srihari Veeraraghavan, Andrew G Nicholson, Christopher P Denton, Andrew Leask, Jeremy D Pearson, Carol M Black, Kenneth I Welsh, Roland M du Bois

**Affiliations:** 1 Interstitial Lung Disease Unit, Royal Brompton Hospital, Imperial College of Science, Technology and Medicine, Emmanuel Kaye Building, 1B Manresa Road, SW3 6LR, London, UK; 2 Division of Academic Rheumatology, Royal Free Hospital, London, U.K; 3 Centre for Cardiovascular Biology and Medicine, Guy's, King's, and St. Thomas' School of Biomedical Sciences, King's College London, UK; 4 Division of Respiratory Diseases, University of Siena, Siena, Italy; 5 MRC Clinical Science Centre, Hammersmith Campus, Imperial College London, UK; 6 Dept of Pathology, Royal Brompton Hospital, London, UK

## Abstract

**Background:**

Transforming growth factor beta (TGFβ), a multifunctional cytokine, plays a crucial role in the accumulation of extracellular matrix components in lung fibrosis, where lung fibroblasts are considered to play a major role. Even though the effects of TGFβ on the gene expression of several proteins have been investigated in several lung fibroblast cell lines, the global pattern of response to this cytokine in adult lung fibroblasts is still unknown.

**Methods:**

We used Affymetrix oligonucleotide microarrays U95v2, containing approximately 12,000 human genes, to study the transcriptional profile in response to a four hour treatment with TGFβ in control lung fibroblasts and in fibroblasts from patients with idiopathic and scleroderma-associated pulmonary fibrosis. A combination of the Affymetrix change algorithm (Microarray Suite 5) and of analysis of variance models was used to identify TGFβ-regulated genes. Additional criteria were an average up- or down- regulation of at least two fold.

**Results:**

Exposure of fibroblasts to TGFβ had a profound impact on gene expression, resulting in regulation of 129 transcripts. We focused on genes not previously found to be regulated by TGFβ in lung fibroblasts or other cell types, including *nuclear co-repressor *2, *SMAD specific E3 ubiquitin protein ligase 2 *(*SMURF2*), *bone morphogenetic protein 4*, and *angiotensin II receptor type 1 *(*AGTR1*), and confirmed the microarray results by real time-PCR. Western Blotting confirmed induction at the protein level of AGTR1, the most highly induced gene in both control and fibrotic lung fibroblasts among genes encoding for signal transduction molecules.

Upregulation of AGTR1 occurred through the MKK1/MKK2 signalling pathway. Immunohistochemical staining showed AGTR1 expression by lung fibroblasts in fibroblastic foci within biopsies of idiopathic pulmonary fibrosis.

**Conclusions:**

This study identifies several novel TGFβ targets in lung fibroblasts, and confirms with independent methods the induction of angiotensin II receptor type 1, underlining a potential role for angiotensin II receptor 1 antagonism in the treatment of lung fibrosis.

## Background

Transforming Growth Factor beta (TGFβ) is a multifunctional cytokine that regulates a variety of physiological processes, including cell growth and differentiation, extracellular matrix production, embryonic development and wound healing [1]. Altered expression of TGFβ plays a crucial role in organ fibrosis, hypertrophic scarring, cancer, autoimmune and inflammatory diseases [2].

In the lung, TGFβ is consistently linked with progressive fibrosis [3-5]. Increased expression of TGFβ has been reported in a variety of fibrotic lung diseases [6,7,3], including idiopathic pulmonary fibrosis (IPF), a relentlessly progressive fibrotic lung disease with a median survival from diagnosis of only two years [8], and pulmonary fibrosis associated with systemic sclerosis, one of the leading causes of death in scleroderma patients [9]. Animal models also support a central role played by TGFβ in lung fibrosis. Intra-tracheal adenovirus-mediated TGFβ gene transfer causes severe lung fibrosis extending to the periphery of the lungs [5]. Mice lacking alphavbeta 6, an integrin which is crucial to the release of active TGFβ from latent extracellular complexes, develop lung inflammation but are strikingly protected from bleomycin-induced lung fibrosis [10]. IL-13 overexpression induces lung fibrosis which is mediated via TGF-β1 induction and activation [11]. Experimental inhibition of TGFβ with neutralizing antibodies, soluble receptors, or gene transfer of the TGFβ inhibitor Smad7, inhibits fibrosis in animal models [12-14].

Lung fibroblasts are the main cell type responsible for excessive extracellular matrix synthesis and deposition in fibrosing lung disorders [15]. TGFβ modulates fibroblast function through several mechanisms, including induction of extracellular matrix protein synthesis and inhibition of collagen degradation [1]. However, knowledge of TGFβ targets in adult lung fibroblasts is still limited to a small number of genes. Oligonucleotide array technology allows the simultaneous assessment of thousands of genes providing a global gene expression profiling of the response to a stimulus. The response to TGFβ has been investigated using oligonucleotide microarrays in keratinocytes [16] as well as in dermal [17] and in a human fetal lung fibroblast line [18], but not in primary human adult lung fibroblasts. Fibroblastic responses are likely to vary with the origin and developmental state of the cells [19], and a detailed study of TGFβ responses in adult lung fibroblasts is needed to gain further insights into the fibroproliferative process in the lung.

We therefore quantified gene expression by oligonucleotide microarrays of adult lung fibroblasts (derived from biopsies of normal and both idiopathic and scleroderma-associated pulmonary fibrosis) in response to TGFβ, and identified several novel TGFβ targets among the wide variety of genes regulated by this cytokine. Of these, we particularly focused on *angiotensin II receptor type 1*, the most highly TGFβ-induced gene among those encoding for signal transduction molecules.

## Methods

### Cell culture

Primary adult lung fibroblasts were cultured from three control samples (unaffected lung from patients undergoing cancer-resection surgery) and from open-lung biopsy samples of lung fibrosis patients, three with idiopathic pulmonary fibrosis (IPF) [8] and three with pulmonary fibrosis associated with the fibrotic disease systemic sclerosis [9]. Independent reviews of the clinical (SV, ER) and histopathologic diagnosis (AGN) were performed. All the idiopathic pulmonary fibrosis biopsies were characterized by a usual interstitial pneumonia pattern (UIP), whereas all of the scleroderma-associated pulmonary fibrosis were classified as non-specific interstitial pneumonia (NSIP) [8]. Verbal and written consent was given by all subjects; authorization was given by the Royal Brompton Hospital Ethics Committee. Fibroblast culture conditions were as previously described [20]. At confluence, lung fibroblasts (all between passages 4–5) were serum-deprived for 16 hours, and exposed to either 4 ng/ml of activated TGF-β1 (R&D Systems) or serum-free culture medium for four hours. The concentration and time point of TGFβ used in our experiments was determined from ongoing studies within our laboratory, in which a 4 hour treatment with TGFβ 4 ng/ml was found to show significant induction of selected known direct TGFβ target genes, including CTGF.

### RNA isolation and gene array analysis

At the end of the treatment period with or without TGFβ, total RNA was harvested (Trizol, Life Technologies), quantified, and integrity was verified by denaturing gel electrophoresis.

Preparation of RNA samples for chip hybridization followed Affymetrix (Affymetrix, Santa Clara, California) protocols. Each RNA sample derived from an individual fibroblast line was hybridized on a separate microarray chip. Hybridization of cRNA to Affymetrix human U95Av2 chips, containing approximately 12,000 well characterized human genes, signal amplification and data collection were performed using an Affymetrix fluidics station and chip reader, following Affymetrix protocol. Scanned files were analyzed using Affymetrix Version 5.0 software (MAS5). Chip files were analyzed by scaling to an average intensity of 150 per gene, as recommended by Affymetrix. Reproducibility was assessed using two pairs of RNA samples from the same control line, TGFβ-treated/untreated; the concordance correlation coefficients were of 0.979 and 0.983, respectively.

TGFβ response was analyzed by using a combination of the MAS5 Affymetrix change algorithm and of ANOVA models. According to Affymetrix criteria, in each TGFβ-treated/medium only pair, genes were defined as differentially regulated (either up or down) by TGFβ only when identified as significantly increased (I) or decreased (D) as determined by the Affymetrix change algorithm, with a change p value<0.001, and were detected as Present (according to the "absolute call"obtained by an Affymetrix algorithm) at least in the samples with the highest count (i.e. medium only in the case of D and TGFβ in the case of I). Genes were defined as TGFβ-responsive in normal human lung fibroblasts when they fulfilled all of the following three conditions: a) they were detected as TGFβ-regulated by Affymetrix criteria (see above) in at least two of the three control pairs; b) they showed a mean fold change after TGFβ of at least 2 (or lower than 0.5) in control fibroblasts; c) either a two-way ANOVA including only control fibroblasts detected a significant (p < 0.05) increase or decrease in control fibroblasts after TGFβ or they were also found to be responsive in at least four of the six fibrotic fibroblast lines and a significant effect (p < 0.05) of treatment (with TGFβ) was detected by a repeated measure ANOVA model including all the samples and adjusting for individual samples, disease, and interaction between treatment and disease. All statistical analyses were performed on log transformed data to reduce inequalities of variance. Thus, the latter ANOVA model could detect genes which were equally up- or down-regulated in normal and fibrotic fibroblasts, taking advantage of the larger number of samples, while the first model (equivalent to a paired t test) could detect changes possibly occurring in controls but not in fibrotic cell lines.

Except for unknown genes, all gene symbols and names are given according to the nomenclature proposed by the Human Genome Organization (HUGO) Gene Nomenclature Committee.

### Real time-PCR

Real time PCR (RT-PCR) was performed to confirm selected novel TGFβ targets in lung fibroblasts. Adult lung fibroblast lines [three control and three fibrotic (IPF)] were treated with or without TGFβ (4 ng/ml) for four hours. Total RNA was isolated from treated and untreated samples using Trizol (Life Technologies) and the integrity of the RNA was verified by gel electrophoresis. Total RNA (1 microgram) was reverse transcribed in a 20 μl reaction volume containing oligonucleotide dTs (dT_18_) and random decamers (dN_10_) using M-MLV reverse transcriptase (Promega) for 1 hour at 37°C. The cDNA was diluted to 100 μl with DEPC-treated water and 1 μl was used per real-time PCR reaction. A set of eight standards containing a known concentration of target amplicon was made by PCR amplification, isolation by gel electrophoresis through a 2% agarose gel followed by gel purification using QIAquick PCR purification spin columns (Qiagen). The concentration of the amplicon was measured by spectrophotometry and diluted in DEPC-treated water containing transfer RNA (10 μg/ml) to make standards of 10 fold dilutions from 100 pg/ μl to 0.01 fg/ μl. The target was measured in each sample and standard by real-time PCR using FastStart DNA Master SYBR Green (Roche Applied Science) as described by the manufacturer, in half the reaction volume (10 μl). Samples and standards were amplified for 30 to 40 cycles with the appropriate primers (Molecular Biology Unit, KCL School of Biological Sciences) at least in duplicate. The amount of target in the sample in picograms was read from the standard curve and values were normalised to 28S ribosomal RNA (pg of target/pg of 28S ribosomal-RNA). The oligonucleotide primer sequences are listed (5'-3'): *angiotensin II receptor type1 *(*AGTR1*) primers: forward TGC TTC AGC CAG CGT CAG TT and reverse GGG ACT CAT AAT GGA AAG CAC; *SMAD specific E3 ubiquitin protein ligase 2 *(*SMURF2*): forward AAC AAG AAC TAC GCA ATG GGG and reverse GTC CTC TGT TCA TAG CCT TCT G; *nuclear receptor co-repressor 2 *(*NCOR2*): forward CAG CAG CGC ATC AAG TTC AT and reverse GTA ATA GAG GAC GCA CTC AGC; *bone morphogenetic protein 4 *(*BMP4*) primers: forward CTA CTG GAC ACG AGA CTG GT and reverse GAG TCT GAT GGA GGT GAG TC.

The results were analyzed using Student's paired t-test after logarithmic transformation, and statistical significance was taken as a p value of <0.05.

### Western blot analysis of TGFβ-induction of angiotensin II receptor 1

Lung fibroblasts were grown to confluence in DMEM with 10% FCS. At confluence, lung fibroblasts (all between passages 2–5) were serum-deprived overnight, and exposed to either 4 ng/ml of activated TGF-β1 (R&D Systems) or serum-free culture-medium with the addition of 0.1% BSA for 24 hours. To determine the signalling pathways through which TGFβ induces AGTR1, lung fibroblasts were treated with specific inhibitors 30 minutes before treatment with TGFβ. These included the dual MKK1/MKK2 inhibitor U0126 (10 μM) and predominant MKK1 inhibitor PD98059 (50 μM), known to inhibit MKK2 only weakly [21], as well as the p38 MAPK inhibitor SB 202190 (30 μM). Cell layer lysates were examined. Cell protein (10 μg/sample) was heated to 99°C for 5 min, loaded into sample wells, resolved on a 12% tricine SDS-polyacrylamide gel (Novex, San Diego, CA), and run at 120 V for 2 h. The separated proteins were transferred onto nitrocellulose membranes at 30V for 90 minutes. Membranes were blocked by incubation for one hour with 5% non-fat milk in phosphate buffered saline (PBS) containing 0.1% Tween 20. They were then washed and incubated overnight at 4°C in a 1:500 dilution of rabbit anti-angiotensin II receptor 1 polyclonal antibody (Santa Cruz Biotechnology), followed by a three-time wash in PBS and incubation in 1:1000 goat anti-rabbit biotinylated IgG (Vector Laboratories, Peterborough, UK) for 60 min at room temperature. Membranes were washed three times in PBS, and the signal was amplified/detected by using the ECL protocol as described by the manufacturer (Amersham plc, Little Chalfont, UK). Films were analysed by laser scanning densitometry on an Ultrascan XL (LKB-Wallac, UK). Data were analyzed by using Student's paired t test after log transformation and a p value<0.05 was considered significant.

### Immunohistochemistry

The distribution of staining for AGTR1 was assessed by immunohistochemistry in surgical lung biopsies from four patients with idiopathic pulmonary fibrosis (IPF), meeting the diagnostic criteria of the American Thoracic Society/European Respiratory Society Consensus Classification [8], and in control biopsies (normal periphery of resected cancer) from three patients undergoing cancer resection surgery. Paraffin-embedded sections were dewaxed with xylene, hydrated and heated in the microwave at 120 degrees for 30 minutes in citrate buffer (10 mM pH 6.0).

Slides were then briefly rinsed in PBS, blocked with 10% normal goat serum for 20', incubated with rabbit polyclonal anti-human AGTR1 antibody (N-10, 1:50, Santa Cruz Biotechnology, Santa Cruz, Calif) for one hour at room temperature. After washing with PBS, sections were incubated with biotinylated goat anti-rabbit IgG diluted in PBS (1:200) for 30 minutes, rinsed, and finally incubated with Vectastain Elite STR-ABC reagent (Vector Laboratories) for 30 minutes. After washing, sections were visualized using 3-amino-9-ethylcarbazole chromogen and H_2_O_2 _as substrate (SK-4200; Vector Laboratories). Sections were then washed in tap water, counterstained with Carrazzis hematoxylin, and mounted with Gelmount (Biomeda, Foster City, CA) for examination using an Olympus BH-2 photomicroscope. Controls included an exchange of primary antibodies with goat matched antibodies. To confirm staining specificity, sections were also incubated with either nonimmune rabbit IgG control or secondary antibody only.

## Results

### Microarray analysis of TGFβ-response in primary adult lung fibroblasts

According to the criteria outlined in the methods, a four hour treatment with TGFβ was found to regulate 129 transcripts in human lung fibroblasts. TGFβ-responsive transcripts included genes with roles in gene expression, matrix formation, cytoskeletal remodelling, signalling, cell proliferation, protein expression and degradation, cell adhesion and metabolism. A complete list of TGFβ-regulated genes is provided (see Additional file 1). The complete set of gene array data has been deposited in the Gene Expression Omnibus database with GEO serial accession number GSE1724 .

We did not observe a substantial degree of difference in the response to TGFβ between the two fibrotic groups (idiopathic pulmonary fibrosis and scleroderma-associated pulmonary fibrosis) and control lung fibroblasts. Once the criteria outlined in the methods section and the p-value for interaction with treatment had been taken into account, there were no significant differences in the response to TGFβ among the three groups except for two genes, KIAA0261 (probe N: 40086_at), an unknown gene more upregulated in IPF (median fold change 2.2) than in scleroderma-associated pulmonary fibrosis (1.5) and in controls (1.3), and BTG1 (probe N: 37294_at), which was only slightly more downregulated in scleroderma-associated pulmonary fibrosis (fold change:0.4) than in IPF (0.6) and in controls (0.7). As both the number of genes and the magnitude of the differences were minimal, they were not considered meaningful and were not investigated further. Among genes responding significantly to TGFβ in control lung fibroblasts, as assessed by ANOVA analysis, none changed in opposite directions in either of the fibrotic groups. All the genes that responded significantly in the control group alone, were also TGFβ-responsive when analysis was extended to include the fibrotic cell lines. Furthermore, none of these genes responded differently to TGFβ between the two fibrotic groups, which are thus presented together in Tables 1 and 2.

**Table 1:** Transcription factor genes regulated by TGFβ in control and fibrotic lung fibroblasts (LF)

Gene Symbol	Affymetrix Probe N	Control LF*	Fibrotic LF*	Gene name
BHLHB2	40790_at	6.0	5.1	basic helix-loop-helix domain containing, class B, 2
CBFB	41175_at	2.9	2.8	core-binding factor, beta subunit
EGR2	37863_at	52.0	3.3	early growth response 2 (Krox-20 homolog, Drosophila)
ETV6	38491_at	2.0	2.6	ets variant gene 6 (TEL oncogene)
FOXO1A	40570_at	3.8	6.0	forkhead box O1A (rhabdomyosarcoma)
JUNB	2049_s_at	3.7	4.2	jun B proto-oncogene
JUNB	32786_at	4.4	3.0	jun B proto-oncogene
LRRFIP1	41320_s_at	2.1	1.5	leucine rich repeat (in FLII) interacting protein 1
MKL1	35629_at	2.7	2.6	megakaryoblastic leukemia (translocation) 1
MSC	35992_at	2.4	1.7	musculin (activated B-cell factor-1)
NCOR2	39358_at	2.2	2.2	nuclear receptor co-repressor 2
NPAS2	39549_at	2.4	3.1	neuronal PAS domain protein 2
NR2F2	39397_at	0.4	0.5	nuclear receptor subfamily 2, group F, member 2
NRIP1	40088_at	2.3	1.8	nuclear receptor interacting protein 1
RUNX1	393_s_at	2.3	2.6	runt-related transcription factor 1 (aml1 oncogene)
RUNX1	39421_at	3.1	2.3	runt-related transcription factor 1 (aml1 oncogene)
RUNX1	943_at	2.2	2.7	runt-related transcription factor 1 (aml1 oncogene)
SKI	41499_at	2.5	2.1	v-ski sarcoma viral oncogene homolog (avian)
SMURF2	33354_at	2.2	2.2	E3 ubiquitin ligase SMURF2
SRF	1409_at	2.1	1.9	serum response factor
SRF	40109_at	2.2	2.0	serum response factor
TCF21	37247_at	0.2	0.4	transcription factor 21
TCF8	33439_at	2.8	1.8	transcription factor 8 (represses interleukin 2 expression)
TIEG	224_at	2.2	2.1	TGFB inducible early growth response
TIEG	38374_at	3.2	2.7	TGFB inducible early growth response
ZFP36L2	32587_at	0.3	0.4	zinc finger protein 36, C3H type-like 2
ZFP36L2	32588_s_at	0.3	0.3	zinc finger protein 36, C3H type-like 2
ZNF365	35959_at	14.2	2.5	zinc finger protein 365

*Mean fold change in mRNA abundance in TGFβ treated/untreated control and fibrotic lung fibroblasts (LF), respectively. Fibrotic lung fibroblast ratios represent the average values of idiopathic and scleroderma-associated pulmonary fibrosis lung fibroblasts.

**Table 2:** TGFβ-regulated signalling and ECM/cytoskeletal genes in control and fibrotic lung fibroblasts

Gene Symbol	Affymetrix Probe N	Control LF*	Fibrotic LF*	Gene name
**Signal transduction**				
ACVR1	39764_at	2.2	1.7	activin A receptor, type I
ADM	34777_at	0.3	0.4	adrenomedullin
AGTR1	346_s_at	3.8	3.2	angiotensin II receptor, type 1
AGTR1	37983_at	5.1	5.9	angiotensin II receptor, type 1
BDKRB2	39310_at	0.4	0.4	bradykinin receptor B2
BMP4	1114_at	0.2	0.2	bone morphogenetic protein 4
BMP4	40333_at	0.1	0.3	bone morphogenetic protein 4
DYRK2	40604_at	3.0	3.0	dual-specificity tyrosine-(Y)-phosphorylation regulated kinase 2
DYRK2	760_at	2.9	3.3	dual-specificity tyrosine-(Y)-phosphorylation regulated kinase 2
DYRK2	761_g_at	3.3	2.2	dual-specificity tyrosine-(Y)-phosphorylation regulated kinase 2
MLP	36174_at	2.4	1.7	MARCKS-like protein
PLK2	41544_at	0.4	0.6	polo-like kinase 2 (Drosophila)
RRAD	1776_at	3.0	5.2	Ras-related associated with diabetes
RRAD	39528_at	3.6	5.1	Ras-related associated with diabetes
SMAD3	38944_at	0.4	0.4	SMAD, mothers against DPP homolog 3 (Drosophila)
SMAD7	1857_at	2.3	2.2	SMAD, mothers against DPP homolog 7 (Drosophila)
SOCS1	41592_at	0.1	0.1	suppressor of cytokine signaling 1
SPRY2	33700_at	2.0	1.8	sprouty homolog 2 (Drosophila)
STK38L	32182_at	3.7	3.8	serine/threonine kinase 38 like
TGFBR3	1897_at	0.3	0.5	transforming growth factor, beta receptor III (betaglycan)
TNFRSF1B	1583_at	0.4	0.6	tumor necrosis factor receptor superfamily, member 1B
TNFRSF1B	33813_at	0.4	0.4	tumor necrosis factor receptor superfamily, member 1B
TSPAN-2	35497_at	4.2	5.0	tetraspan 2
**Extracellular matrix remodelling/Cytoskeletal**				
COL4A1	39333_at	2.2	2.0	collagen, type IV, alpha 1
COMP	40161_at	2.7	5.3	cartilage oligomeric matrix protein
COMP	40162_s_at	5.0	18.9	cartilage oligomeric matrix protein
CTGF	36638_at	4.8	6.1	connective tissue growth factor
CYR61	38772_at	4.4	3.5	cysteine-rich, angiogenic inducer, 61
ELN	31621_s_at	4.9	3.7	elastin
ELN	39098_at	8.4	11.6	elastin
PLAUR	189_s_at	2.7	2.8	plasminogen activator, urokinase receptor
PLOD2	34795_at	2.5	1.8	procollagen-lysine, 2-oxoglutarate 5-dioxygenase 2
SERPINE1	38125_at	3.7	4.0	serine (or cysteine) proteinase inhibitor, clade E, member 1
SERPINE1	672_at	6.0	5.5	serine (or cysteine) proteinase inhibitor, clade E, member 1
TIMP3	1034_at	2.0	1.5	tissue inhibitor of metalloproteinase 3
TIMP3	1035_g_at	2.4	1.6	tissue inhibitor of metalloproteinase 3
TPM1	36790_at	2.3	1.7	tropomyosin 1 (alpha)
TPM1	36791_g_at	2.7	2.1	tropomyosin 1 (alpha)
TPM1	36792_at	2.5	2.0	tropomyosin 1 (alpha)

Mean fold change in mRNA abundance in TGFβ treated/untreated control and fibrotic lung fibroblasts (LF), respectively. Fibrotic lung fibroblast ratios represent the average of idiopathic and scleroderma-associated pulmonary fibrosis lung fibroblasts.

For the purpose of this study, we will concentrate on genes involved in transcriptional regulation, cytoskeletal/extracellular matrix organization, and signal transduction (Tables 1 and 2).

#### Control of transcription

TGFβ regulated a wide array of transcription factors (Table 1), including the known TGFβ target *JUNB*. Other TGFβ targets in lung fibroblasts identified by this study included Smad co-activators *RUNX1 *and *CBFB*, recently implicated in the targeted subnuclear localization of TGFβ-regulated Smads [22,23]. Transcriptional regulators involved in cell cycle control/cell differentiation induced by TGFβ included *FOXO1A*, *NPAS2*, and *TIEG *(*TGFβ-inducible early growth response*), while *ZFP36L2*, a zinc finger transcription factor linked to cell proliferation induction, was repressed by TGFβ. *Serum response factor *(*SRF*) and *MKL1 *were also induced by TGFβ. Transcriptional repressors induced by TGFβ included *Ski*, which together with *Sno *interacts with Smad molecules to inhibit transcription and may contribute to terminating TGFβ response [24] and *TCF8*, a previously reported TGFβ target in fetal lung fibroblasts [18]. Other transcriptional co-repressors upregulated by TGFβ were nuclear co-repressors *NCOR2 *(or *SMRT*) and *BHLHB2*, which repress transcription by recruiting histone deacetylases [25], and *musculin *(*MSC*).

#### Cytoskeletal/Extracellular matrix organization

Most genes in this category were known TGFβ targets. As expected, transcripts involved in promoting extracellular matrix formation and cell adhesion such as *connective tissue growth factor *(*CTGF*) were upregulated, while we observed inhibition of *bone morphogenetic protein 4 *(*BMP4*), a member of the TGFβ superfamily whose activity has recently been shown to be inhibited by CTGF through direct binding [26].

TGFβ also induced matrix genes including *elastin *(*ELN*), collagens (*COL4A1*), *plasminogen activator inhibitor *(*PAI1 *or *SERPINE1*) and *PLOD2*, an enzyme which stabilizes collagen cross-links (Table 2). *Tissue inhibitor of matrix metalloproteinase 3 *(*TIMP3*) was upregulated by TGFβ. Genes involved in cytoskeletal organization induced by TGFβ included known target *tropomyosin *(*TPM1*). Interestingly, *smoothelin*, a smooth muscle gene recently reported to be highly induced by TGFβ in fetal lung fibroblasts [18], was also induced by TGFβ in this study, but at a slightly lower fold ratio than that chosen for the selection criteria (1.8).

#### Control of signal transduction

Among signalling molecules (Table 2), known targets included upregulation of *SMAD7 *and downregulation of *SMAD3 *[18,16]. Novel targets in lung fibroblasts included *SMURF2*, a recently identified E3 ubiquitin ligase, which negatively regulates TGFβ signalling by targeting both TGFβ receptor-Smad7 complexes and Smad2 for ubiquitin-dependent degradation [27,28]. At the investigated timepoint, TGFβ downregulated the accessory receptor *betaglycan*, a membrane anchored proteoglycan which increases the affinity between TGFβ and type I and II receptors. Interestingly, TGFβ upregulated *activin A type I receptor*, a receptor for TGFβ family member activin, whose stimulation induces fibroblast-mediated collagen gel contraction [29]. Members of the Ras family of GTPases, *ARHB *and *RADD *(Ras-related GTP-binding protein), involved in cytoskeleton remodelling, were also upregulated by TGFβ. TGFβ also induced *Dickkopf1 *(*DKK1*), a potent inhibitor of Wnt/beta-catenin signalling.

Of particular interest was the novel observation that TGFβ upregulated *angiotensin II receptor 1 *(*AGTR1*) in lung fibroblasts; conversely, the gene encoding for vasodilatory peptide *adrenomedullin *(*ADM*) was inhibited by TGFβ.

### Validation of selected TGFβ-induced genes by real time RT-PCR

**Figure 1:**
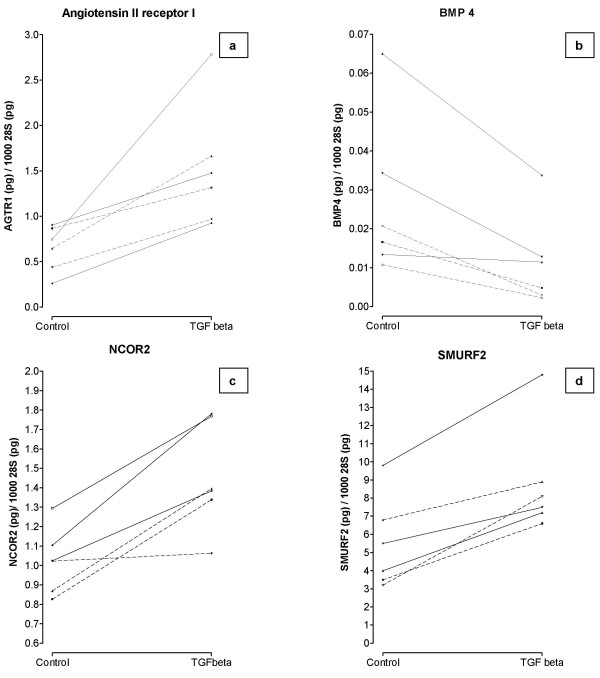
*Independent verification of microarray results by measurement of gene expression with real time-PCR*. TGFβ treatment (4 ng/ml) for four hours induces expression of mRNA for angiotensin receptor 1 (panel a), nuclear receptor co-repressor 2 (*NCOR2*) (panel c) and SMURF2 (panel d) as well as inhibition of bone morphogenetic protein 4 (panel b) in three control lung fibroblast cell lines (dashed lines) and three fibrotic lung fibroblasts (solid lines).

Several of the genes regulated by TGFβ confirmed previously published findings, thus validating our methods, including *JUN-B*, *SMAD7*, *connective tissue growth factor*, *elastin*, and *SERPINE1 *[17,18,16,30]. To further consolidate our analysis, we selected a small group of novel TGFβ targets to be confirmed by RT-PCR in both control and fibrotic lung fibroblasts. These novel fibroblast TGFβ-responsive genes included potential key candidates in the regulation by TGFβ of lung tissue fibrosis and included *angiotensin II receptor type 1 *(*AGTR1*), *SMURF2*, a gene involved in terminating TGFβ signalling, *NCOR2*, a transcriptional co-repressor and *BMP4*, a member of the TGFβ family. Compared to untreated samples, we confirmed that TGFβ upregulated *AGTR1 *(ratio = 2.4; p = 0.002), *SMURF2*, (ratio = 1.8, p = 0.003), *NCOR2 *(ratio 1.4; p = 0.004), and downregulated *BMP4 *(ratio = 0.4; p = 0.009), with no difference in the response between control and fibrotic fibroblasts (Figure 1).

### Induction of angiotensin II receptor type 1 by TGFβ

**Figure 2:**
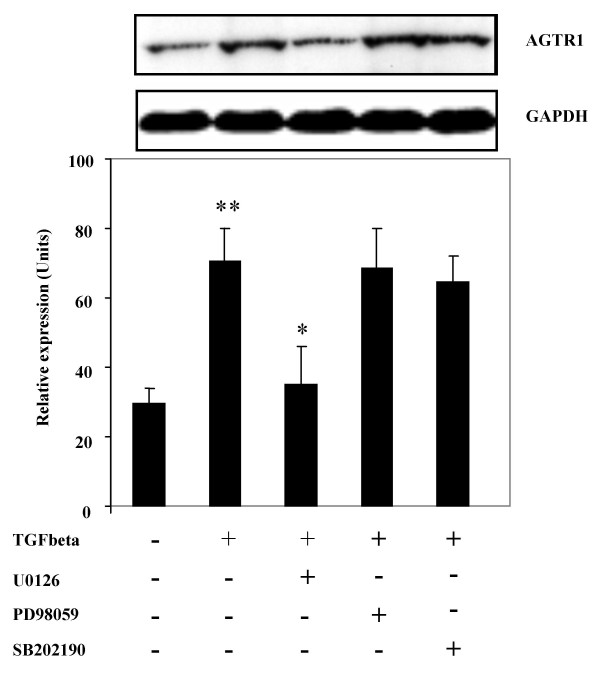
*TGFβ treatment induces angiotensin II receptor 1 (AGTR1) protein expression in adult lung fibroblasts; the induction is mediated by MKK1/MKK2*. Representative Western Blot (top) and average values (± SD) of angiotensin II receptor type 1 protein expression in lung fibroblasts treated with TGFβ (4 ng/ml)with or without 1/2 hour pre-incubation with of one the following signalling inhibitors: U0126, PD98059, SB202190. A 24 hour treatment with TGFβ induced an upregulation of AGTR1 protein (mean: 2.4 fold, **p < 0.001, Student's paired t-test). The induction of AGTR1 by TGFβ was specifically blocked by MKK1/MKK2 inhibitor U1026 (*p < 0.01 compared with TGFβ-induced AGTR1, Student's paired t-test), but not by predominant MKK1 inhibitor PD98059 or p38 inhibitor SB202190). The results are representative of three independent experiments on both control and fibrotic cell lines. As a loading control, Western analysis with an anti-GAPDH antibody was also performed.

We focused on AGTR1 protein because, as shown by microarray analysis, it was the most highly TGFβ-induced gene among signaling molecules in both control and fibrotic fibroblasts (Table 2). To verify whether *AGTR1 *mRNA upregulation corresponded to an increase in protein levels, we performed Western analysis on primary human adult lung fibroblasts exposed to TGFβ or medium alone in serum-free conditions for 24 hours. The intensity of the angiotensin II receptor 1 immunoreactive band was significantly increased in TGFβ-treated fibroblasts compared to those treated with medium alone (2.4 fold; p < 0.001) (Figure 2). To identify the signalling pathways through which TGFβ induces AGTR1, we evaluated whether the ability of TGFβ to induce AGTR1 expression in lung fibroblasts was blocked by specific signaling pathway inhibitors. A 30 minute preincubation with the dual MKK1/MKK2 inhibitor U0126 significantly inhibited TGFβ induction of AGTR1 protein (p < 0.01), whereas predominant MKK1 inhibitor PD98059 and p38 MAPK inhibitor SB202190 had no significant effect (Figure 2).

### AGTR1 expression in idiopathic pulmonary fibrosis lung biopsies

**Figure 3:**
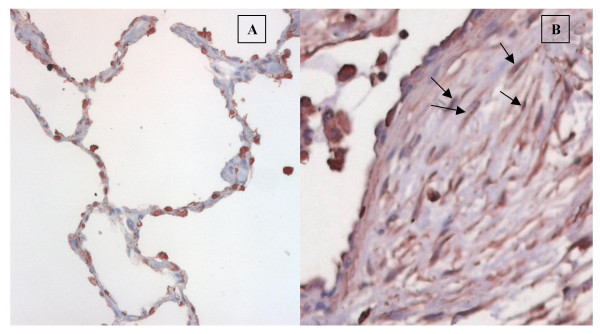
*Angiotensin II receptor 1 staining in lung biopsies from control patients (A) and from patients with idiopathic pulmonary fibrosis (B)*. Immunohistochemistry for the angiotensin II receptor 1 (AGTR1), counterstained with haematoxylin. AGTR1 positive staining is seen in alveolar macrophages, in epithelial cells and in fibroblastic foci (arrows) in usual interstitial pneumonia biopsies (panel B). Epithelial cells and alveolar macrophages express AGTR1 in control lung biopsies (panel A).

We assessed staining for AGTR1 in lung biopsies from four patients with idiopathic pulmonary fibrosis and compared it to that of three control lungs. In particular we aimed to evaluate AGTR1 staining in fibroblastic foci, aggregates of fibroblasts/myofibroblasts in close contact with alveolar epithelial cells. Both in control and in idiopathic pulmonary fibrosis lung biopsies, AGTR1 immunoreactivity was observed in alveolar epithelial cells and alveolar macrophages. In addition, the fibroblasts within the fibroblastic foci present in idiopathic pulmonary fibrosis biopsies stained positive for the receptor (Figure 3).

## Discussion

In this study we report, for the first time, the transcriptional profile in response to TGFβ in adult primary human lung fibroblasts both from control and from fibrotic lungs. Our analysis of the response to TGFβ focused on TGFβ gene targets involved in transcription and signalling, identifying a series of genes previously unknown to respond to TGFβ in lung fibroblasts. These included angiotensin II receptor 1, providing further insights into links between TGFβ and angiotensin in the pathogenesis of fibrosis [31,32].

Although gene expression profiling in response to TGFβ has been investigated previously, earlier work has been confined to skin fibroblasts [17], keratinocytes [16], and a human fetal lung cell line [18], which is likely to respond differently to TGFβ from the adult lung fibroblast. Our data cannot be directly compared with the fetal lung fibroblast profiling because of methodological disparities, chiefly due to differences in the timing of the RNA collection. However, even restricting the comparison to results obtained at similar time points, we found a significant dissimilarity. Among transcription factors, only *JUNB *and *TCF8 *were upregulated by TGFβ both in fetal [18] and in adult lung fibroblasts, while all others differed between the two cell types. Interestingly, in this study, TGFβ caused an induction of both *MKL1 *and *serum response factor*, while neither were upregulated in fetal lung fibroblasts. The recently reported cooperation between these two transcription factors in determining smooth muscle cell differentiation [33] suggests that they may play a similar role in lung fibroblasts and suggests differences between fetal and adult lung fibroblasts in the transcriptional programs involved in the TGFβ-induced acquisition of the myofibroblastic phenotype.

In this study, we did not observe a substantial difference in the response to TGFβ between lung fibroblasts from two patterns of fibrotic lung disease and control lung fibroblasts. *In vivo *heterogeneity between interstitial lung fibroblasts may occur in fibrotic and normal lung, obscuring the demarcation between normal and abnormal phenotypes, when cell lines are isolated using standard techniques [34,35]. This may explain discrepancies among studies on growth rate and resistance to apoptosis in fibroblasts derived from fibrotic lungs [34,36]. In particular, the fibroblasts/myofibroblasts forming the fibroblastic foci, observed to be linked to disease progression [37], could differ from the remaining fibroblasts found in the interstitium. The issue of sampling a population of homogeneous lung fibroblasts will be the subject of further investigation by using laser microdissection techniques targeting fibroblastic foci coupled with new technologies to amplify RNA from limited quantities of tissue [38]. Further, it is possible that the absence of striking differences in the response to TGFβ between disease groups and controls is due to a loss of the pro-fibrotic phenotype in vitro, even though the gene expression patterns of different passages of the same fibroblast line have been observed to cluster together, indicating that the *in vitro *phenotypes are stable through several passages in culture [19]. Further, we ensured that RNA was extracted from all fibroblast lines at comparable passages. Thus, even though our study cannot exclude the presence of subtle differences in the response to TGFβ, we have observed that, overall, fibrotic lung fibroblasts retain the capacity to respond to TGFβ, which could therefore be targeted by pharmacological means.

Among the novel TGFβ targets identified by microarray analysis in lung fibroblasts, we focused our attention on the induction of *angiotensin II receptor type 1 *(*AGTR1*), as its involvement is likely to significantly amplify the pro-fibrotic actions of TGFβ. The ligand for this receptor is angiotensin II, a vasoactive peptide which has been linked to fibrogenesis in the kidney and in the heart [39,40]. Recent studies have indicated that a local renin-angiotensin system could also be involved in the development of lung fibrosis [41,42]. Elevated angiotensin converting enzyme levels have been found in bronchoalveolar lavage (BAL) fluid from patients with idiopathic pulmonary fibrosis [41]. Compared to controls, lung fibroblasts from patients with idiopathic pulmonary fibrosis produce higher levels of angiotensin II, shown to induce apoptosis in alveolar epithelial cells through AGTR1 [31,43]. Blockade of angiotensin II or of AGTR1 attenuates lung collagen deposition in animal models of lung fibrosis [42,32]. Interestingly, the modulation of AGTR1 could be cell specific, as suggested by the report that TGFβ reduces AGTR1 expression in cardiac fibroblasts [44].

In addition to Smad molecules, the classic signalling pathway used by TGFβ family members, TGFβ also signals through the mitogen-activated protein kinase (MAPK) signalling pathways [16]. In this study, TGFβ was found to induce AGTR1 via mitogen-activated protein kinase kinase (MKK1/MKK2). The finding that the MKK1/MKK2 inhibitor U0126, but not the MKK1 inhibitor PD98059, was able to suppress TGFβ-induced AGTR1 expression, suggests that both MKK1 and MKK2 must be antagonized in order to inhibit transcription.

The functional effects of AGTR1 stimulation in lung fibroblasts are only partially known. Although two isoforms of angiotensin II receptor exist, AGTR1 and AGTR2, the effects described so far of angiotensin II on lung fibroblasts are ascribed to the type 1 receptor. AGTR1 has been found to mediate mitogenesis in human lung fibroblasts [45] and extracellular matrix synthesis in lung [46] as well as in cardiac and dermal fibroblasts [47]. Whereas angiotensin II is known to induce TGFβ [46], the regulation of AGTR1 by TGFβ has not, to our knowledge, been previously reported in lung fibroblasts. Our data support the concept of a positive feed back loop by which TGFβ potentiates the pro-fibrotic actions of angiotensin II by increasing AGTR1 expression, providing a mechanism for the attenuation of the proliferative response to angiotensin II by TGFβ blockade [45]. Thus, cooperation and amplification of pro-fibrotic effects between TGFβ and AGTR1 are likely to be implicated in lung fibrosis. Interestingly, adrenomedullin, a multifunctional vasodilatory peptide that downregulates angiotensin II-induced collagen biosynthesis in cardiac fibroblasts [48], was inhibited by TGFβ, confirming a previous report [49], and suggesting that TGFβ exerts a complex regulation over vasoactive peptides and/or their receptors in lung fibroblasts.

AGTR1 was found to localize to fibroblasts within fibroblastic foci in IPF/UIP biopsies. An increase in AGTR1 staining has been reported in the fibrotic regions surrounding the bronchioles in chronic obstructive pulmonary disease [50]. The finding that AGTR1 localizes to fibroblastic foci in IPF biopsies supports the potential relevance of the angiotensin system in this disease and suggests that the pro-fibrotic role of AGTR1 in IPF is not limited to epithelial cells [31]. Further studies are needed to assess the functional effects of AGTR1 stimulation in lung fibroblasts and to evaluate the biological role of AGTR1 in lung fibrosis.

## Conclusions

Our findings confirm that in response to TGFβ, both control and fibrotic lung fibroblasts are potent effector cells expressing a very wide range of genes that are likely to contribute to the fibrotic process. In particular, we have shown that TGFβ has the capacity to influence the expression of angiotensin II receptor type 1 both at the mRNA and at the protein level. In view of the known induction of TGFβ by angiotensin II [45], our findings support the existence of a self-potentiating loop between TGFβ and angiotensin II, resulting in the amplification of the pro-fibrotic effects of both systems. Future treatment strategies could be based on the disruption of such interactions.

## Authors' contributions

EAR participated in the design and interpretation of the study, carried out the cell culture work and participated in the microarray work, performed immunohistochemistry staining, and drafted the manuscript. DJA participated in the design and coordination of the study and in the preparation of the manuscript, SH performed the RT-PCR assays, XSW carried out the Western Blot analysis, PS performed the statistical analysis and participated in the interpretation of results and preparation of the manuscript, GBG participated in the microarray work, AUW participated in the interpretation of results, SV participated in cell line selection and clinical characterization, AGN reviewed fibrotic lung biopsies and interpreted immunohistochemistry staining, CD and CMB contributed towards the overall organizational setup for the study of lung fibroblast lines and participated in the interpretation of results, AL and JDP participated in the preparation of the manuscript, KIW conceived of the study and participated in the design, RdB participated in study design, interpretation and coordination. All authors read and approved the final manuscript.

## Supplementary Material

Additional File 1 Complete list of genes regulated by a four hour treatment with TGFβ in control and fibrotic fibroblasts This data set contains all the genes up- or down-regulated by a four hour treatment with TGFβ (according to the criteria described in the methods) in control and fibrotic lung fibroblasts. Fibrotic lung fibroblast fold ratios are the average of the fold ratios for lung fibroblasts from idiopathic pulmonary fibrosis and pulmonary fibrosis associated with systemic sclerosis. Genes are sub-grouped into functional classes. Affymetrix probe set numbers, approved gene symbols, gene names and GenBank accession numbers are provided in the table. Click here for file
